# Spatiotemporal diffusion of influenza A (H1N1): Starting point and risk factors

**DOI:** 10.1371/journal.pone.0202832

**Published:** 2018-09-04

**Authors:** Ana Carolina Carioca da Costa, Cláudia Torres Codeço, Elias Teixeira Krainski, Marcelo Ferreira da Costa Gomes, Aline Araújo Nobre

**Affiliations:** 1 National Institute of Women, Children and Adolescents Health Fernandes Figueira, Department of Clinical Research, Oswaldo Cruz Foundation, Rio de Janeiro, Brazil; 2 Scientific Computing Program, Oswaldo Cruz Foundation, Rio de Janeiro, Brazil; 3 Federal University of Paraná, Paraná, Brazil; 4 Norwegian University of Science and Technology, Trondheim, Norway; Columbia University, UNITED STATES

## Abstract

Influenza constitutes a major challenge to world health authorities due to high transmissibility and the capacity to generate large epidemics. This study aimed to characterize the diffusion process of influenza A (H1N1) by identifying the starting point of the epidemic as well as climatic and sociodemographic factors associated with the occurrence and intensity of transmission of the disease. The study was carried out in the Brazilian state of Paraná, where H1N1 caused the largest impact. The units of spatial and temporal analysis were the municipality of residence of the cases and the epidemiological weeks of the year 2009, respectively. Under the Bayesian paradigm, parametric inference was performed through a two-part spatiotemporal model and the integrated nested Laplace approximation (INLA) algorithm. We identified the most likely starting points through the effective distance measure based on mobility networks. The proposed estimation methodology allowed for rapid and efficient implementation of the spatiotemporal model, and provided evidence of different patterns for chance of occurrence and risk of influenza throughout the epidemiological weeks. The results indicate the capital city of Curitiba as the probable starting point, and showed that the interventions that focus on municipalities with greater migration and density of people, especially those with higher Human Development Indexes (HDIs) and the presence of municipal air and road transport, could play an important role in mitigation of effects of future influenza pandemics on public health. These results provide important information on the process of introduction and spread of influenza, and could contribute to the identification of priority areas for surveillance as well as establishment of strategic measures for disease prevention and control. The proposed model also allows identification of epidemiological weeks with high chance of influenza occurrence, which can be used as a reference criterion for creating an immunization campaign schedule.

## Introduction

Influenza poses a major challenge to world health authorities due to high transmissibility and capacity to generate major epidemics. From an epidemiological point of view, influenza epidemics and pandemics are associated with changes in the structure of society that favor the spread of new strains in specific ecological, social, and spatial contexts [1]. The 2009 influenza A (H1N1) virus emerged from the combination of genetic segments from the human influenza virus, avian influenza virus, and swine influenza virus, and was first identified in April 2009 in Mexico and the United States [2]. The virus spread rapidly across the globe and on June 11, 2009, the World Health Organization (WHO) declared influenza A (H1N1) as a pandemic [2]. In April 2009, preventive actions were initiated to delay entry of the virus to Brazil. In border regions, seaports and airports began to utilize sound alerts and distribute information for the purpose of early identification of symptomatic individuals. Despite these efforts, on July 16, 2009, the Ministry of Health officially announced sustained transmission of the H1N1 virus in the Brazilian territory [3]. The first cases of H1N1 influenza in Brazil were imported from countries that already had sustained transmission of the disease [4]. According to Ministry of Health data, up to the epidemiological week (EW) 47 (11/22/2009 to 11/28/2009), 30,055 cases of Severe Acute Respiratory Infection (SARI) had been registered, with 93% identified as resulting from H1N1 infections. The H1N1 pandemic disproportionately affected the southern and southeast regions of Brazil, with Paraná being the most affected state, accounting for 52% of the total cases reported in the country [5]. Despite low lethality rates, the rapid spread of the disease generated panic in Paraná. As in other parts of the country and the world, preventive measures were taken, such as the closure of schools, restaurants, and public places, in addition to social distancing and changes in hygiene habits [6]. Although the 2009 pandemic provided extensive knowledge and experience for public health professionals, the mechanism responsible for the spread of influenza is not yet fully understood [7]. The present study modeled the spatiotemporal evolution of the 2009 influenza A (H1N1) epidemic in Paraná, using efficient computational methods to identify climatic and sociodemographic factors associated with the chance of H1N1 introduction and, once introduced, the intensity of spread within the city. The model was further used to identify the most-likely starting points of H1N1 diffusion into the state of Paraná. These results will contribute to evaluation of timely control measures and decision making by public health authorities.

## Materials and methods

### Study area

**Fig 1:**
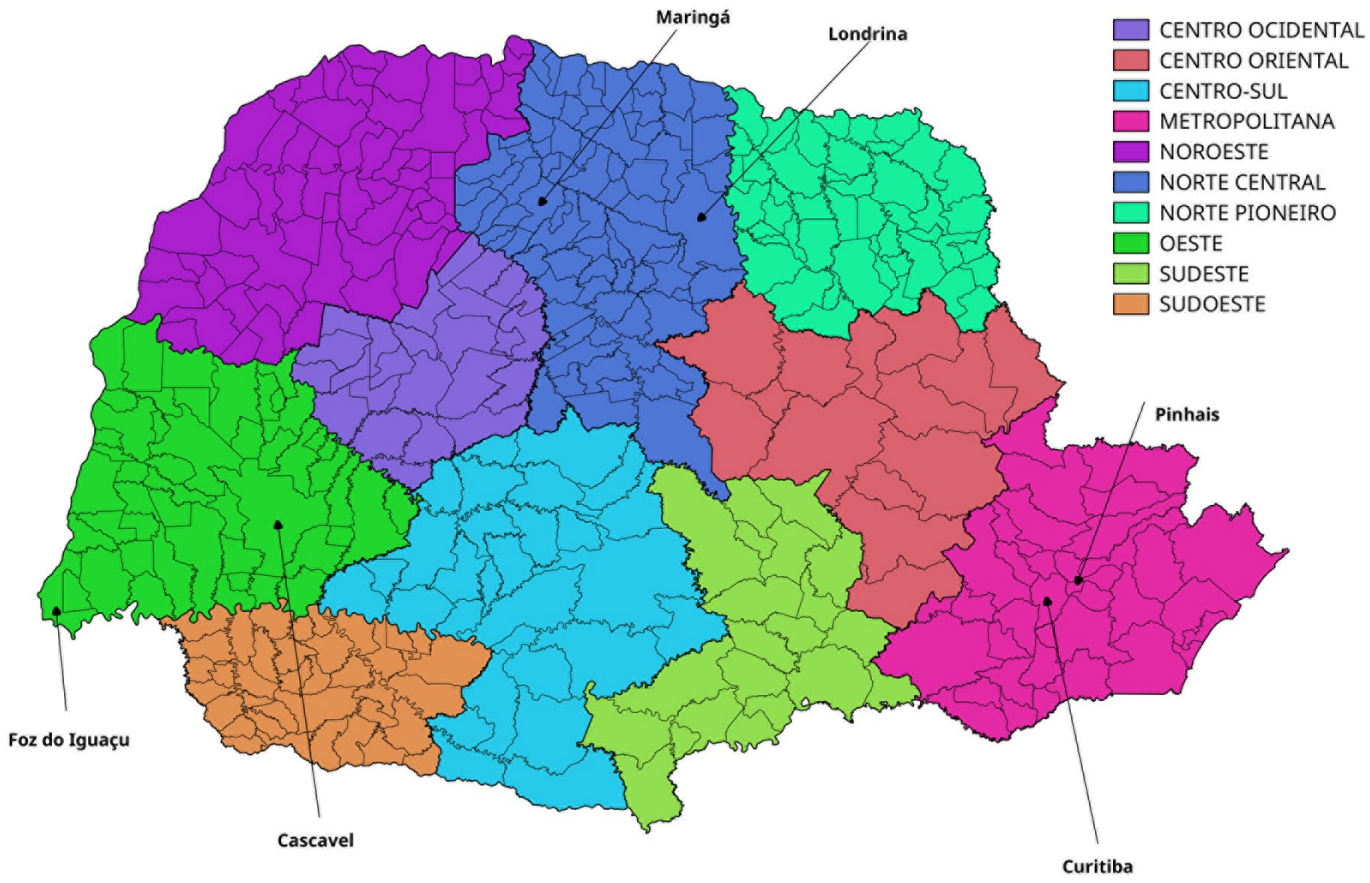
Map of Paraná. Map of the state of Paraná broken down by mesoregion.

The study area is Paraná State in the southern region of Brazil. Paraná (25°25’21”S, 52°02’15”W) borders the Brazilian states of São Paulo, Santa Catarina, and Mato Grosso do Sul, and is also bordered by Paraguay and Argentina, thus serving as gateway for entry to Brazil for residents of these countries (Fig 1). The average temperature in the state is 18.5°C, and the climate is divided into two regimes: a tropical regime that predominates in the North, West, and Coastal areas with average temperatures of 22°C, and a subtropical or temperate regime in the mid-southern area with average temperatures between 10°C and 22°C. Tourism is one of the main sectors of the economy, and Paraná has the 5th highest Human Development Index (HDI) in Brazil. The population of Paraná is about four times less than in the state of São Paulo, with 11 million inhabitants; approximately 2 million reside in Curitiba, the state capital and most populous municipality. Iguazu falls, at the border between Argentina, Brazil and Paraguay, is also an important touristic region attracting people around the world.

### Epidemiological Data

In Paraná, active surveillance of suspected cases of influenza was initiated in April 5, 2009. Any medical doctor diagnosing a suspected case should notify to the National Notification System, SINAN [8]. Suspected cases were subsequentely confirmed or discarded based on the results of laboratory tests or by ascertaining an epidemiological link between the suspected case and a previously confirmed one. Laboratory testing was carried out in 27% of the suspected cases. In July 2009, there was a recommendation for restricting notification to patients with severe acute respiratory illness (SARI) only. However, in Paraná, this recommendation was not followed, as the proportion of mild cases remained high throughout the epidemic [5].

Time series of influenza cases were created by summing the number of weekly confirmed and autochthonous cases, from April 5 to September 26, 2009. We used the municipalities of residence as the spatial unit for analysis, and epidemiological week of symptom onset as the temporal unit for analysis. On some occasions, there was no record for initial day of symptoms. In these cases, missing data were estimated using imputation technique, and the corresponding value for first day of symptoms was calculated as 2.76 days prior to the day-of-report (the mean time between onset of symptoms and notification of illness), following the estimation of Codeço et al. [5].

### Sociodemographic and Climate Data

The climate variables used in the study were precipitation, temperature (minimum and maximum), relative humidity, and altitude. Rainfall data were obtained through the National Water Agency (ANA), based on 515 rainfall stations. The data for minimum temperature, maximum temperature and humidity were obtained from the National Institute of Meteorology (INMET), the Paraná Agronomic Institute (IAPAR), and the Paraná Meteorological System (SIMEPAR). Temperature data (in degrees Celsius) were obtained from 113 weather stations and humidity data (expressed as percentage) were based on data from 99 stations. Altitude data (in meters) were obtained from the Brazilian Institute of Geography and Statistics (IBGE) and are reported for the seat of each municipality.

All stations had some missing meteorological data, for reasons such as malfunction of measuring instruments, reorganization of station networks, or occasional interruptions of automatic stations, among others. To address this problem, we used the CIDW multiple imputation technique (modified correlation coefficient with inverse distance weighting) for precipitation data, as described in Suhaila et al. [9]. For other missing climatic variables, we used the regEM method (regularized EM algorithm) described in Schneider [10].

The unit for spatial analysis was the municipality of residence for cases, thus it was necessary to use meteorological information on the same scale. To this end we calculated mean values for each municipality. We accomplished this by interpolating data from the meteorological stations to a regular grid (1 km) covering the entire state of Paraná. We then calculated the weekly average of each variable by municipality considering the number of pixels of the regular grid contained in each municipality. The interpolation was carried out through generalized additive models (GAMs) [11] using the latitude, longitude, and altitude of the points as covariates. We also evaluated effects of the climate variables precipitation, temperature, and humidity on the incidence of H1N1 infection with a one week lag; this time lag is consistent with the incubation period of the virus in humans.

The sociodemographic variables used in this study were: poverty rate, human development index (HDI), population density (inhabitants per km^2^), density of physicians per 1,000 inhabitants, pendular migration, and presence of primary means of transport (intercity and city bus, boat, and plane). The poverty rate was obtained from the Parana Institute of Economic and Social Development (IPARDES), and HDI data through the United Nations Development Program (UNDP), a body linked to the United Nations. Both data refer to the year 2010. Influenza notification data were obtained through the Ministry of Health, specifically in the Health Information Booklets of the Department of Informatics of SUS (DATASUS) for the year 2009. Population density, pendular migration, and means of transportation offered by each municipality were obtained through IBGE. Pendular migration, defined in this work as the expected number of individuals commuting between municipalities for work or study, was extracted from the microdata of IBGE’s 2010 census. Information regarding means of transportation were extracted from the 2009 Basic Municipal Information Survey [12].

### Ethics statement

This study was approved in accordance with ethical standards and guidelines of Oswaldo Cruz Foundation, CAAE: 31006714.3.0000.5240.

### Spatiotemporal modeling

**Fig 2:**
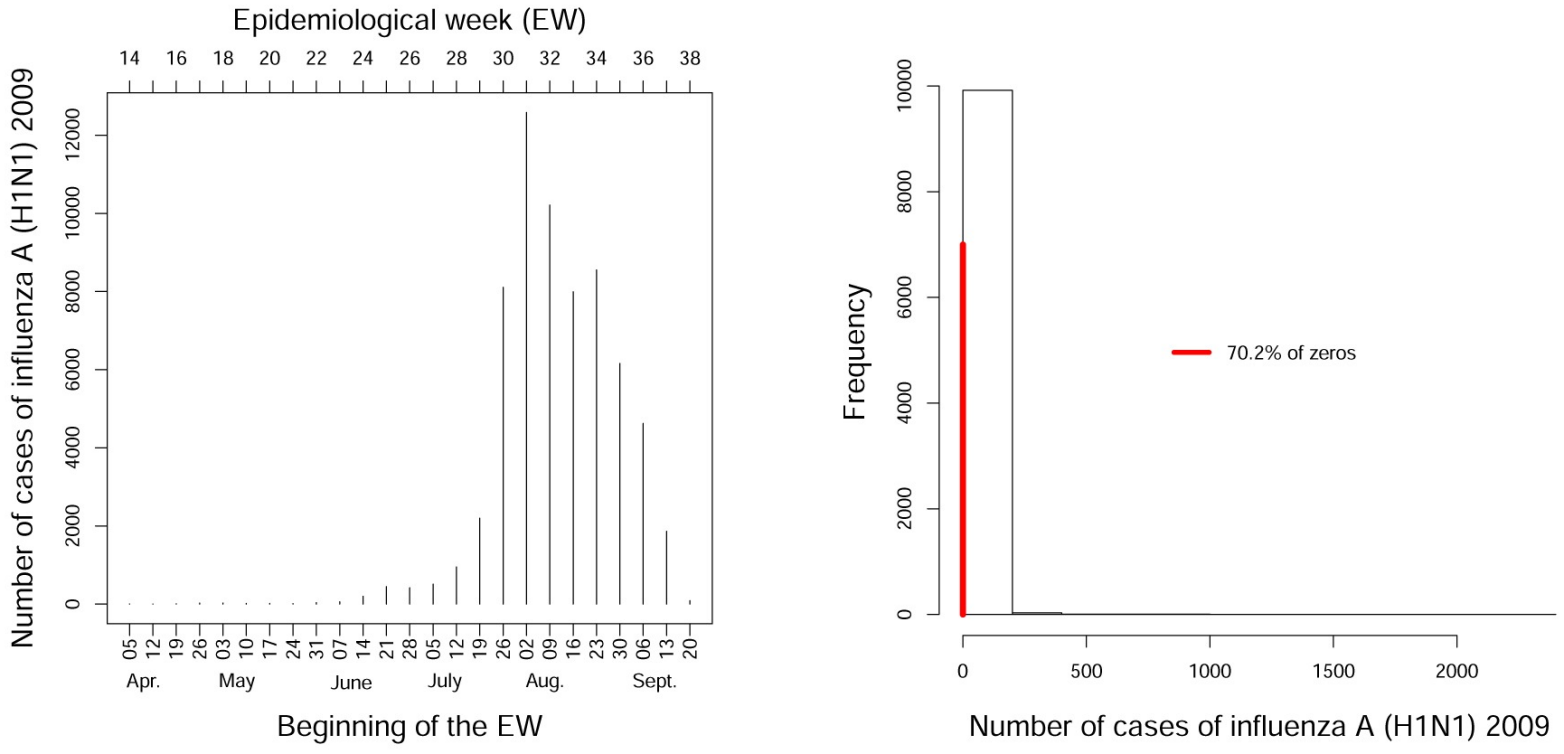
Temporal Series and Histogram. Distribution of the number of confirmed and autochthonous cases of influenza A (H1N1) in Paraná State from epidemiological weeks 14 to 38 (04/05/2009 to 09/26/2009). The red vertical line represents the number of zeros in the data.

Despite the epidemic nature of influenza in Paraná, cases were absent in about 70% of all observations, resulting in an inflation of zeros for the models (Fig 2). From an epidemiological perspective, the distribution of influenza cases can be seen as a manifestation of two processes: (1) occurrence, represented by a binary variable indicating the presence or absence of cases, and (2) intensity, represented by a count variable. The count variable can only be observed when the binary variable indicates presence. From a statistical perspective, two-stage or two-part models (hurdle models) [13] are appropriate for analysis of the process of influenza dissemination.

The number of confirmed and autochthonous cases of influenza A (H1N1) in the epidemiological week *t*, *t* = 1, …, *T*, and municipality *i*, *i* = 1, …, *N* can be zero or a positive number. The occurrence of cases will be defined as

$$\begin{array}{c} z_{it}=\{\begin{array}{c} 1,\;\text{if}\;\text{there}\;\text{are}\;\text{confirmed}\;\text{cases}\;\text{of}\;\text{influenza}\;\text{A}\;\text{(H1N1)}\\ 0,\;\text{if}\;\text{there}\;\text{are}\;\text{no}\;\text{confirmed}\;\text{cases}\;, \end{array} \end{array}$$

and the number of cases reported as

$$\begin{array}{c} y_{it}=\{\begin{array}{c} \text{omitted},\;\text{if}\;\text{there}\;\text{are}\;\text{no}\;\text{cases}\;\text{of}\;\text{influenza}\;\text{A}\;\text{(H1N1)}\\ \text{total}\;\text{number}\;\text{of}\;\text{cases},\;\text{if}\;\text{they}\;\text{occurred}. \end{array} \end{array}$$

The likelihood of this model can be written by the combination of two likelihoods, given by a binomial distribution and a Poisson distribution truncated to zero (ZAP- Zero-Altered Poisson) for *y*_*it*_, i.e.,

$$\begin{array}{c} z_{it}∼\text{Binomial}(1,\pi_{it})\;\text{and}\;y_{it}\;|\;y_{it}>0∼\text{ZAP}(\mu_{it})\;, \end{array}$$

where *π*_*it*_ is the probability of one or more cases of influenza being reported in municipality *i*, epidemiological week *t*, and *μ*_*it*_ is the mean of the truncated Poisson distribution. Given that *y*_*it*_ > 0, the mean *μ*_*it*_ of the truncated Poisson distribution will be modeled as follows:

$$\begin{array}{c} \text{log}(\lambda_{it})=\beta_{0}^{y}+\sum_{l=1}^{L}\beta_{l}^{y}x_{it}^{\left(l\right)}+\sum_{p=1}^{P}\gamma_{pt}^{y}w_{it}^{\left(p\right)}+\xi_{it}\;, \end{array}$$ (1)

where *μ*_*it*_ is defined in terms of rate λ_*it*_ and the expected number of cases of the disease *E*_*it*_ as *μ*_*it*_ = λ_*it*_ *E*_*it*_ for municipality *i* and week *t*. The intercept $\beta_{0}^{y}$ represents the average risk of influenza in the municipalities of Paraná, $\beta^{y}=\left(\beta_{1}^{y},\ldots ,\beta_{L}^{y}\right)$ quantifies the effect of *L* covariates ***x*** = (*x*^(1)^, …, *x*^(*L*)^) and $\gamma_{t}^{y}=\left(\gamma_{1t}^{y},\ldots ,\gamma_{Pt}^{y}\right)$ quantifies the effect of *P* covariates **w** = (*w*^(1)^, …, *w*^(*P*)^), which are assumed to vary in time according to a random first order process defined as $\gamma_{pt}^{y}\;|\;\gamma_{pt-1}^{y}∼\text{Normal}\left(\gamma_{pt-1}^{y},\tau_{\gamma }^{y}\right)$. The risk of influenza A (H1N1) for each municipality in a specific epidemiological week is given by the estimate of λ_*it*_ and the relative risk is obtained by exponentiating the ZAP regression coefficients. Finally, *ξ*_*it*_ is the random spatio-temporal effect that evolves dynamically over time according to a first order autoregressive process—AR(1)—with temporal correlation coefficient *ρ* and spatially structured innovations given by *υ*_*it*_,

$$\begin{array}{ccc} \xi_{it} & = & \rho \xi_{i,t-1}+\upsilon_{it}\;,\;\text{where}\;\left|\rho \right|<1. \end{array}$$

Basically, the random effect ***ξ*** reflects the intuitive notion that the structure of temporal and spatial dependence in a given municipality depends on the spatiotemporal pattern of neighboring municipalities.

Consider a Besag-York-Mollie (BYM) specification [14], in which a spatially structured residue with a zero mean multivariate Normal distribution and covariance matrix **D** is modeled by an intrinsic conditional autoregressive structure (iCAR) such that **D**^−^ = **R**/*σ*^2^, where **R** is determined by the structure of neighboring municipalities. The elements of **R** are given by

$$\begin{array}{c} R_{ij}=\{\begin{array}{c} n_{i},\;\text{se}\;i=j\\ -1\{i∼j\},\;\text{se}\;i\neq j\;, \end{array} \end{array}$$ (2)

where *n*_*i*_ is the number of municipalities bordering the municipality *i*, *i* ∼ *j* indicates that *i* and *j* are neighbors and 1 is the indicator function. Under this specification, the average spatial effect for a given municipality given all other effects is equal to the mean spatial effects for the neighboring regions, and the conditional variance is inversely proportional to the number of neighbors [15]. One limitation of the ICAR model is that the variance *σ*^2^ represents both overdispersion and spatial dependence. In order to avoid this restriction, another specification will be used for the covariance structure of ***υ***, which is based on the generalized inverse of the covariance matrix **D** such that

$$\begin{array}{c} \sigma_{\upsilon }^{2}D^{-}=\left(1-\delta \right)I+\delta R\;, \end{array}$$ (3)

where **I** is the identity matrix, **R** is the intrinsic regression matrix specified in (2) and *δ* ∈ [0, 1] is the parameter that quantifies spatial dependence. In other words, the parameter *δ* represents the weight assigned to the spatially structured component compared to the unstructured term. If *δ* = 0, the specification corresponds to the independent model (**D** = *σ*^2^**I**), and if *δ* = 1, the specification corresponds to the intrinsic autoregressive model (**D** = *σ*^2^**R**^−^). For additional details, see Leroux et al. [16].

The probability of occurrence for influenza *π*_*it*_ will be defined through a logistic link function, and the linear predictor will be the sum of the spatiotemporal effects and covariates. Specifically, the logarithm of chance of occurrence will take the following form:

$$\begin{array}{ccc} \text{log}(\frac{\pi_{it}}{1-\pi_{it}}) & = & \beta_{0}^{z}+\sum_{m=1}^{M}\beta_{m}^{z}x_{it}^{\left(m\right)}+\sum_{r=1}^{R}\gamma_{rt}^{z}w_{it}^{\left(r\right)}+\phi \xi_{it} \end{array}$$ (4)

$$\begin{array}{ccc} \xi_{it} & = & \rho \xi_{i,t-1}+\upsilon_{it}\;, \end{array}$$

where $\beta_{0}^{z}$ is the intercept that quantifies the average chance of disease occurrence in the municipalities of Paraná, and the coefficients ***β***^*z*^ and $\gamma_{t}^{z}$ quantify the effects of covariates. The chance of influenza occurrence for a given municipality and epidemiological week is given by the estimate of *π*_*it*_/1 − *π*_*it*_. If we exponentiate Binomial regression coefficients, the numbers obtained can be interpreted in terms of odds ratios. *ϕ* is the scale parameter for *ξ*_*it*_ which represents the random spatiotemporal effect common in the first component of the model defined in Eq 1. The joint distribution for the random spatiotemporal effects of each process relates to both parts, allowing us to specify a viable model for two distinct processes related to the same phenomenon. The other components assume the same specifications as above. We note that the proposed model allows us to adjust different groups of covariates for each part of the model, that is, it allows us to construct one set of covariates to explain the chance of occurrence of influenza, and another set of covariates to explain the intensity of the disease.

**Fig 3:**
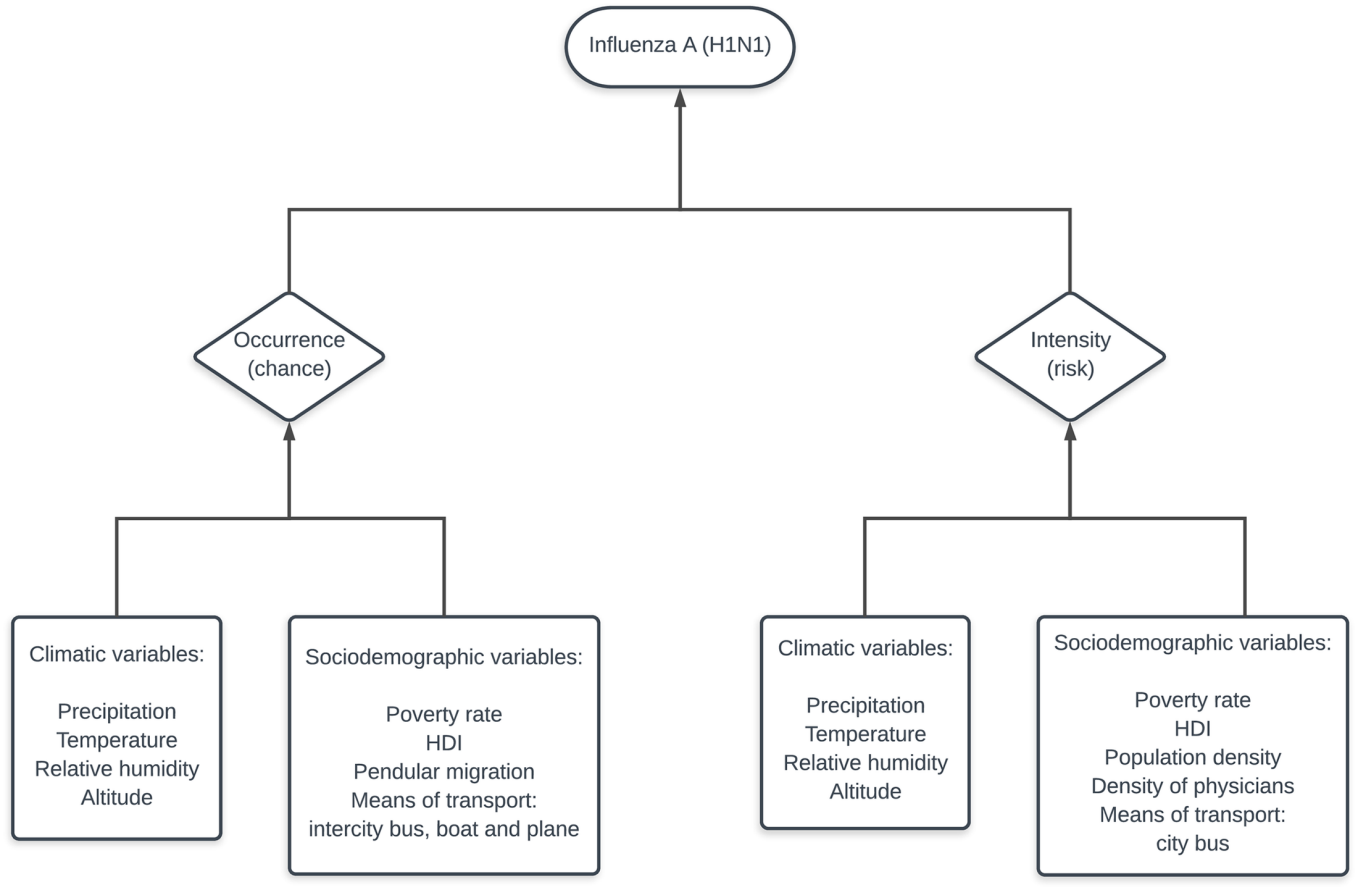
Theoretical model. Climatic and sociodemographic variables used in the theoretical model for the chance of occurrence and risk of influenza A (H1N1).

The sociodemographic and climatic covariates were selected based on a theoretical model of disease occurrence and intensity, as described in Fig 3. Climatic factors such as precipitation, temperature, relative humidity and altitude can impact the occurrence and intensity of influenza transmission, since the virus can “live” for up to 48 hours under favorable weather conditions. Climatic factors may also influence human behavior with regard to contact rate and aggregation patterns. Poverty rate and HDI can be important global determinants of flu occurrence and intensity since they are markers of vulnerabilities that includes health, education and income components. Intercity bus, boat and airplane transportation are supposed to impact the chance of occurrence, since they facilitate the displacement of people between municipalities. Also, pendular migration can increase the occurrence of flu since it favors the disease dissemination. As influenza virus spreads through human contact, population density and bus transportation are potential factors to increase the intensity of transmission. Also, the density of physicians is supposed to impact the intensity of the disease because reflect municipalities’ development and health workforce. Following the notation in Eq 4, *M* = 7 covariates (***x***^(*m*)^) whose effects are fixed in space-time can impact the chance of occurrence: altitude, poverty rate, HDI, pendular migration and intercity bus, boat and plane transportation. According to Eq 1, *L* = 6 covariates (***x***^(*l*)^) are assumed to impact the intensity of the disease: altitude, poverty rate, HDI, population density, density of physicians and city bus transportation. Analogously, *R* = *P* = 3 climatic covariates (***w***^(*r*)^ and ***w***^(*p*)^) which effects are assumed to vary in time can impact both occurrence and intensity: precipitation, temperature and relative humidity. The Pearson correlation coefficients were calculated to test for multicollinearity for both (i.e., occurrence and intensity). Next, we tested for significance of sociodemographic covariates on the occurrence and intensity of influenza excluding correlated covariates. Finally, we tested for effects of conditional climatic variables (i.e., with sociodemographic covariates from the previous step).

The hyperparameter vector of the model is defined as $\psi =\{\rho ,\sigma_{\upsilon }^{2},\tau_{\gamma }^{z},\tau_{\gamma }^{y},\delta ,\phi \}$. From the bayesian point of view, the specification of the model is complete when prior distributions are assigned to the hyperparameters. The following specifications were assumed for the prior distributions of hyperparameters: log((1 + *ρ*)/(1 − *ρ*)) ∼ Normal(0, 6.67), log(*τ*_*υ*_) ∼ logGamma(1, 0.0005), $\text{log}(\tau_{\gamma }^{z})∼\text{logGamma}(1,0.00005)$, $\text{log}(\tau_{\gamma }^{y})∼\text{logGamma}(1,$ 0.00005), log(*δ*/(1 − *δ*)) ∼ Normal(0, 2.22) and *ϕ* ∼ Normal(1, 0.10).

Marginal posterior distributions are not available in closed form. From the bayesian point of view, the most common approach is to make inference through Markov chain Monte Carlo (MCMC) method. However, in complex models, this method can be computationally expensive and difficult to assess the convergence. As an alternative to the MCMC methods, the Integrated Nested Laplace Approximation (INLA) [17] algorithm can be used, which is relatively fast and produces accurate approximations for marginal posterior distributions. In this work, the parametric inference was approximated using INLA. All analyses were performed in R version 3.1.2 [18] using the R-INLA package [19].

### Starting point of the influenza diffusion process

The approach used to identify the start point of the process of spatial diffusion of influenza in Paraná was originally proposed by Brockmann and Helbing [20], and consists of replacing the notion of conventional geographical distance with an effective distance measurement based on networks of mobility. The basic principle is that despite the structural complexity of transport networks, and the geographical distances involved, the dynamical process of contagion is dominated by the set of more probable paths that individuals can take from one place to another, based on the connectivity matrix. Consider the connectivity matrix **P**, where 0 ≤ *P*_*ij*_ ≤ 1 denotes the probability of a person leaving a municipality *i* and arriving in municipality *j*, which is connected to *i*. The effective distance from *j* to *i* can be defined as

$$\begin{array}{c} d_{ij}=\left(1-log\left(P_{ij}\right)\right)\geq 1. \end{array}$$

As discussed in Brockman and Helbing [20], the effective distance is defined based on the logarithm of the transition probability in order to preserve both the additive nature of a distance measure and the multiplicative nature of the probability of traversing a path with multiple steps.

From the definition of effective distance between connected nodes, we can define the effective length λ(Γ) of an ordered path Γ = {*c*_1_, …, *c*_*F*_} as the sum of the effective distance between each step of the path. The effective distance between arbitrary municipalities *j* and *i* in the mobility network is then defined as the length of the shortest route from *j* to *i* as follows

$$\begin{array}{c} D_{ij}=\underset{\Gamma }{\text{min}}\lambda \left(\Gamma \right) \end{array}$$ (5)

Typically, *D*_*ij*_ ≠ *D*_*ji*_. From the perspective of a particular municipality of origin *j*, the set of shortest paths for all other municipalities constitutes the shortest path tree Ψ_*j*_. In other words, Ψ_*j*_ represents the most likely sequence of paths from root municipality *j* to other municipalities. The concept of effective distance reflects the idea that a large flow of people *j* → *i* is equivalent to a highly probable path (i.e., “short” distance), and vice versa. Therefore, if municipality *i* is the source of an infectious pathogen, based on mobility alone municipalities *j* with lower effective distance are expected to be invaded by that pathogen sooner than municipalities with larger effective distance.

In order to assess the municipality(ies) that is(are) more likely to be the starting point(s) for Influenza A (H1N1) introduction in the State of Paraná during the 2009 pandemic, we tested the correlation between the shortest path tree of candidate municipalities against the estimated arrival times in the remaining municipalities. In order to do that, we calculated the shortest path tree Ψ_*j*_ for each of the potential sites of origin of the epidemic and the arrival times of the epidemic. The arrival times in each municipality were defined (using results of the spatiotemporal model described above) as the first epidemiological week in which the probability of having an excess chance of influenza was greater than 0.90. For each candidate city, the correlation coefficients between the effective distances and the times of arrival of the epidemic were calculated. This approach should result in greater correlation when a particular municipality *j* in fact represents the starting point of the epidemic.

The connectivity matrix is defined here based on the pendular migration network described in the Sociodemographic and Climate Data section. The weights of the connectivity matrix are defined as in Eq 5.

## Results

**Fig 4:**
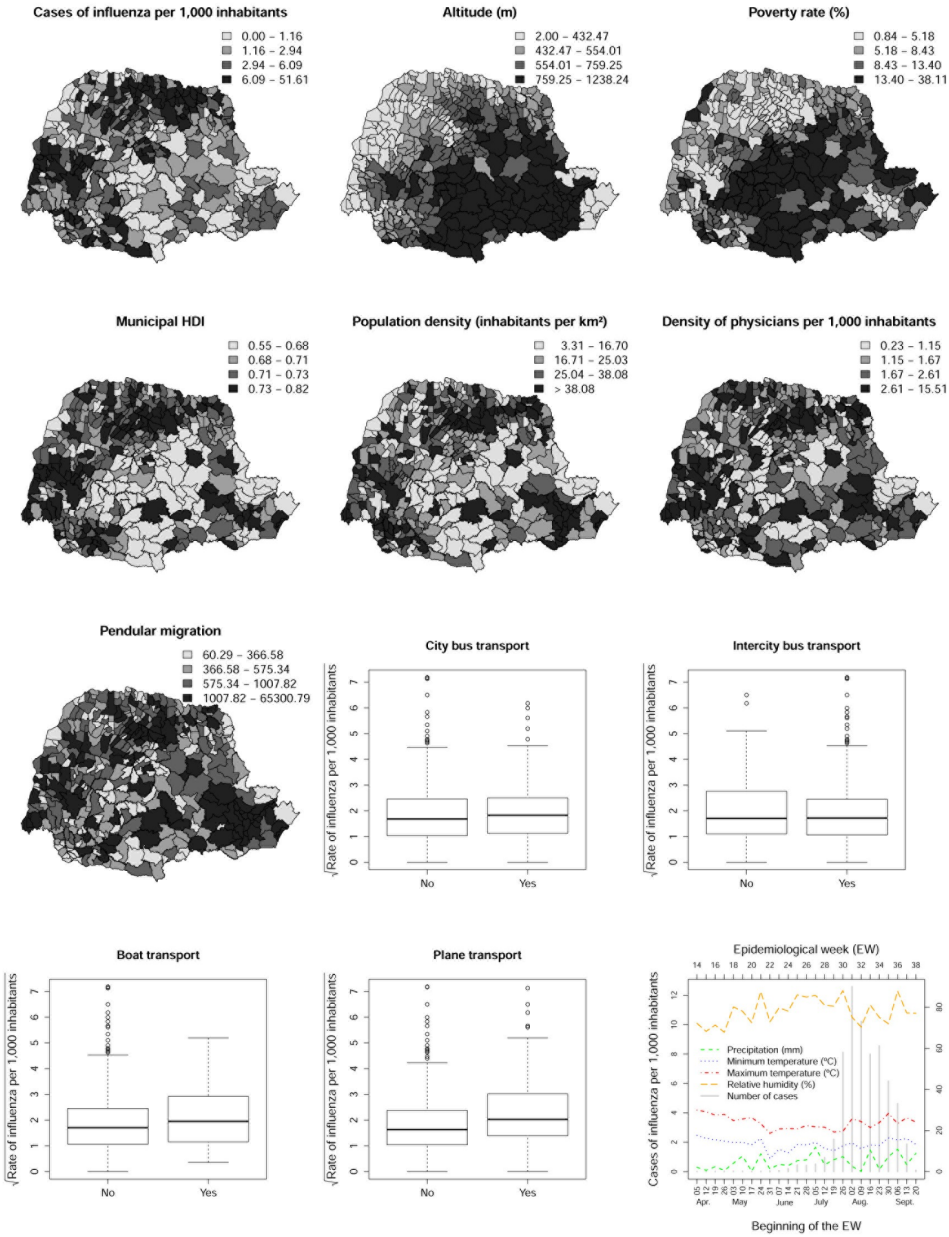
Spatial distribution maps for rate of influenza A (H1N1) and covariates. Map of the spatial distribution of influenza A (H1N1) cases per 1,000 inhabitants with climatic and sociodemographic covariates.

The spatial distribution of influenza A (H1N1) cases per 1,000 people and its relation to sociodemographic covariates is illustrated in Fig 4. During the study period, influenza cases were mainly concentrated in the municipalities within the metropolitan, northern, and western regions of Paraná, where the highest rates of the disease were observed. In general, the maps suggest that higher altitudes and poverty rates may be related to a lower concentration of cases. Municipal HDI, demographic and medical densities, and pendular migration presented heterogeneous spatial distribution patterns compatible with the most affected regions of the state. Higher rates of influenza were observed in municipalities with primary means of transport with the exception of intercity highways, where this relationship was not confirmed. The was no apparent relationship between the total number of cases reported weekly and climate covariates.

Table 1 shows the effects of covariates that were retained in the final spatiotemporal model, expressed in terms of chance ratio and the relative risk of the disease in Paraná. We found that pendular migration, presence of air transport, and HDI significantly contributed to increase the chance of influenza. Population density, municipal road transport, municipal HDI and maximum temperature represented risk factors for the disease intensity. The effect of the covariate maximum lagged temperature of one week (lag 1) was assumed to vary over time, and therefore is omitted from Table 1. The variables minimum temperature, relative humidity, altitude, poverty rate, and medical density were not included in the spatiotemporal model due to multicollinearity. Presence of boat transportation in the municipality, the existence of intercity road transport, and rainfall did not contribute significantly to either the chance of occurrence or intensity of influenza A (H1N1) in Paraná and were also not included in the final regression model.

**Table 1:** Relative risk and chance ratios. Effects of sociodemographic covariates expressed in terms of chance of occurrence of influenza A (H1N1) and the relative risk of disease in Paraná, from April 5 to September 26, 2009, with 95% credible interval.

**Binomial model: occurrence**	
**Effect**	**Odds Ratio (95% CI)**
Pendular migration	1.104 (1.013–1.203)
Plane transport	1.560 (1.379–1.763)
Municipal HDI	1.615 (1.524–1.711)
**ZAP model: intensity**	
**Effect**	**Relative Risk (95% CI)**
Population density	1.171 (1.035–1.340)
City bus transport	3.284 (2.786–3.871)
Municipal HDI	3.558 (3.242–3.906)
Maximum temperature (lag 1)*	—

*The maximum temperature (lag 1) was assumed to vary over time.

**Fig 5:**
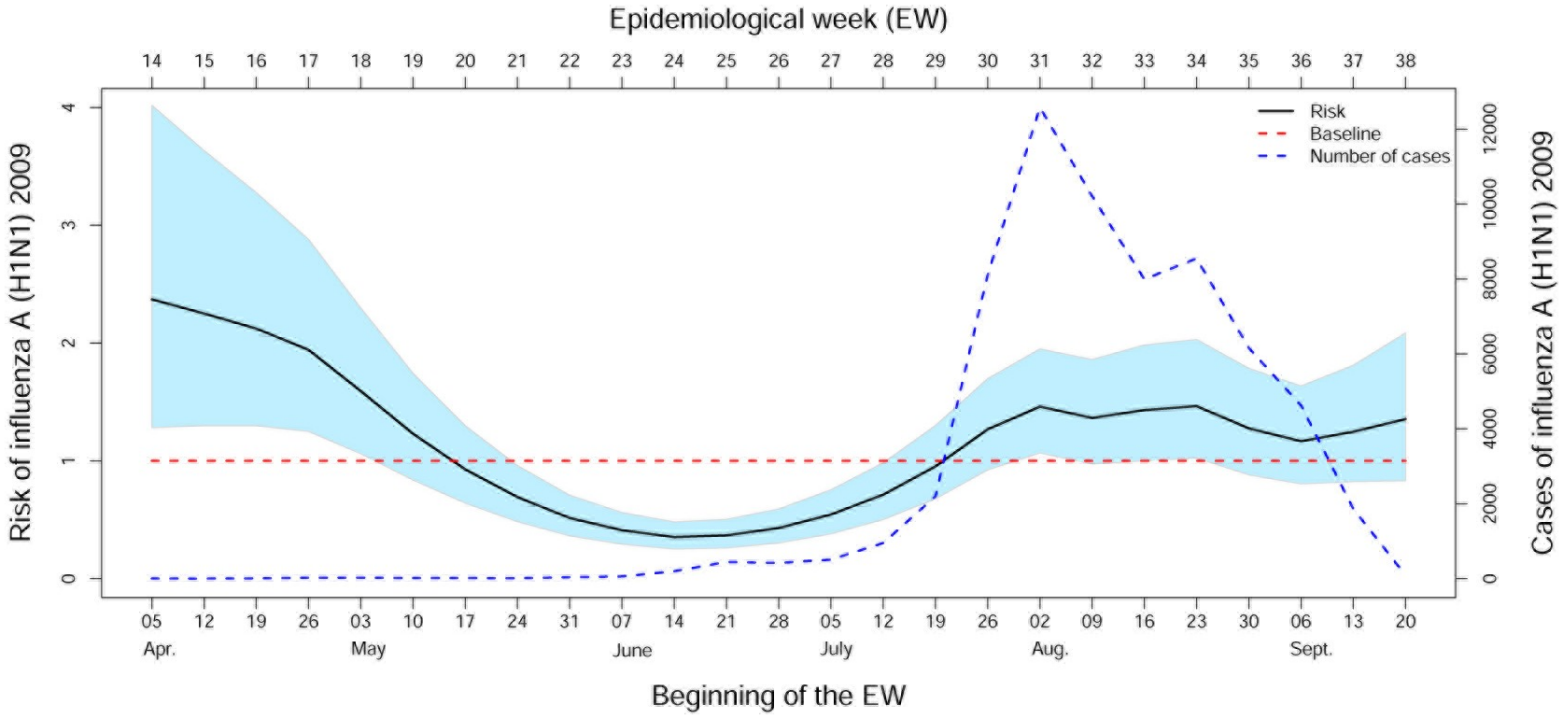
Effect of maximum temperature. Effect of the maximum temperature on the risk of influenza A (H1N1) in Paraná State from April 5 to September 26 in 2009 (corresponding to epidemiological weeks 14 to 38).

The Brazilian influenza A (H1N1) pandemic was divided into two phases: the containment phase and the mitigation phase. The containment phase was the period of introduction of the virus, in which cases of the disease were related to international travel or contact with sick persons who had traveled internationally. This phase was from EW 16 (04/19) to EW 28 (07/12) in 2009, a period that preceded the declaration of sustained transmission of the virus. The mitigation phase began in EW 29 (07/19). The actions recommended for this phase were aimed at reducing morbidity and mortality due to the disease through the early diagnosis and treatment of cases that presented greater risk for severe outcomes or death. In EWs 14 through 18, the period of introduction of the virus in Brazil, there was a significant increase in the incidence of influenza A associated with increased temperature. However, from EW 21 (06/24) to EW 28 (07/12), the period that preceded the sustained transmission phase, reduced temperature favored increased risk of influenza. In general, after the start of the mitigation phase (EW 28) there was no substantial impact of temperature on disease risk (Fig 5).

The spatial correlation parameter, *δ* = 0.965(0.964 − 0.965), suggests a high degree of spatial dependence for dissemination of pandemic influenza in Paraná. Analogously, the value of the temporal correlation coefficient *ρ* = 0.509(0.508 − 0.511) confirms the short-term persistence of the number of confirmed and autochthonous cases of influenza A (H1N1). An effect significantly different from zero was also observed for the parameter that relates the effects of the spatiotemporal interaction of both processes, such that *ϕ* = 0.211(0.208 − 0.216) indicates that the occurrence of cases of the disease also have a heterogeneous spatiotemporal pattern.

**Fig 6:**
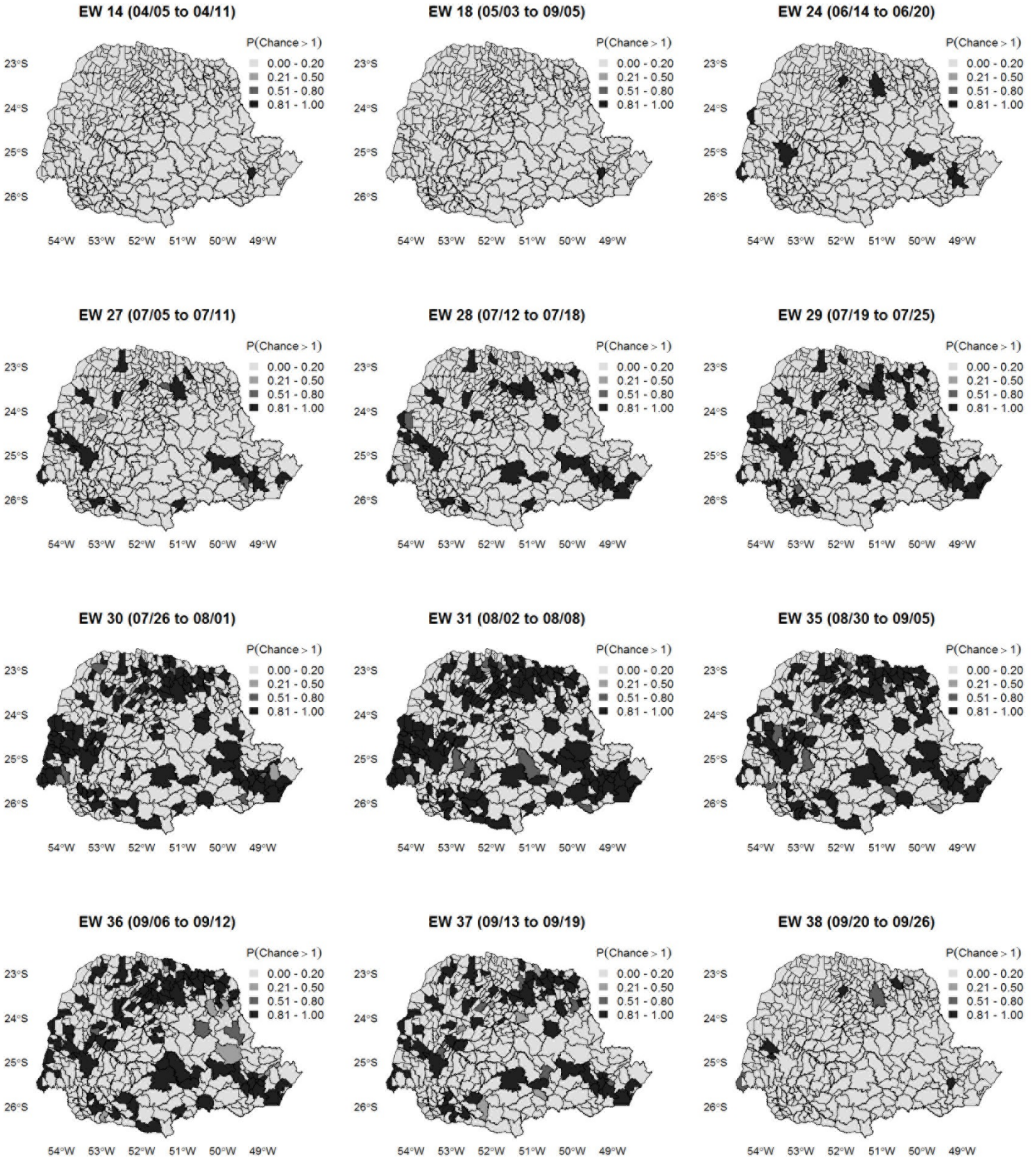
Influenza A (H1N1) chance probability map. Map of the posterior probabilities of influenza A (H1N1) chance exceeds 1 over selected epidemiological weeks in the state of Paraná.

Fig 6 shows the map of the posterior probabilities of infuenza A (H1N1) chance exceeds 1 for some epidemiological weeks. As we are particularly interested in increased chance, we can visualize the level of uncertainty associated with estimating the chance of influenza when it is greater than 1. The probability maps revealed heterogeneity in the diffusion process of influenza A in Paraná, expliciting underlying geographical differences which caused some municipalities to reach the peak of the epidemic before others. There was high chance of influenza A in the capital Curitiba in the first week of April (EW 14), when the virus was installed in the national territory. From EW 24 (06/14 to 06/20), outbreaks of virus began to spread, concentrating mainly on sites that were geographically distant but important from a socioeconomic perspective. Beginning with EW 28 (07/12 to 07/18), there was a substantial increase in the probability of occurrence of influenza cases in Paraná as a whole, characterizing the beginning of the sustained transmission period according to an official announcement from the Ministry of Health. The epidemic spread rapidly throughout the state, reaching the peak for chance of occurrence in EW 31 (08/02 to 08/08). Overall, from EW 35 (08/30 to 09/05) onwards, there was a progressive reduction in the chance of influenza occurrence, culminating in EW 38 (09/20 to 09/26) when few municipalities still had a high probability of disease occurrence, mainly those in which the virus was identified precociously.

**Fig 7:**
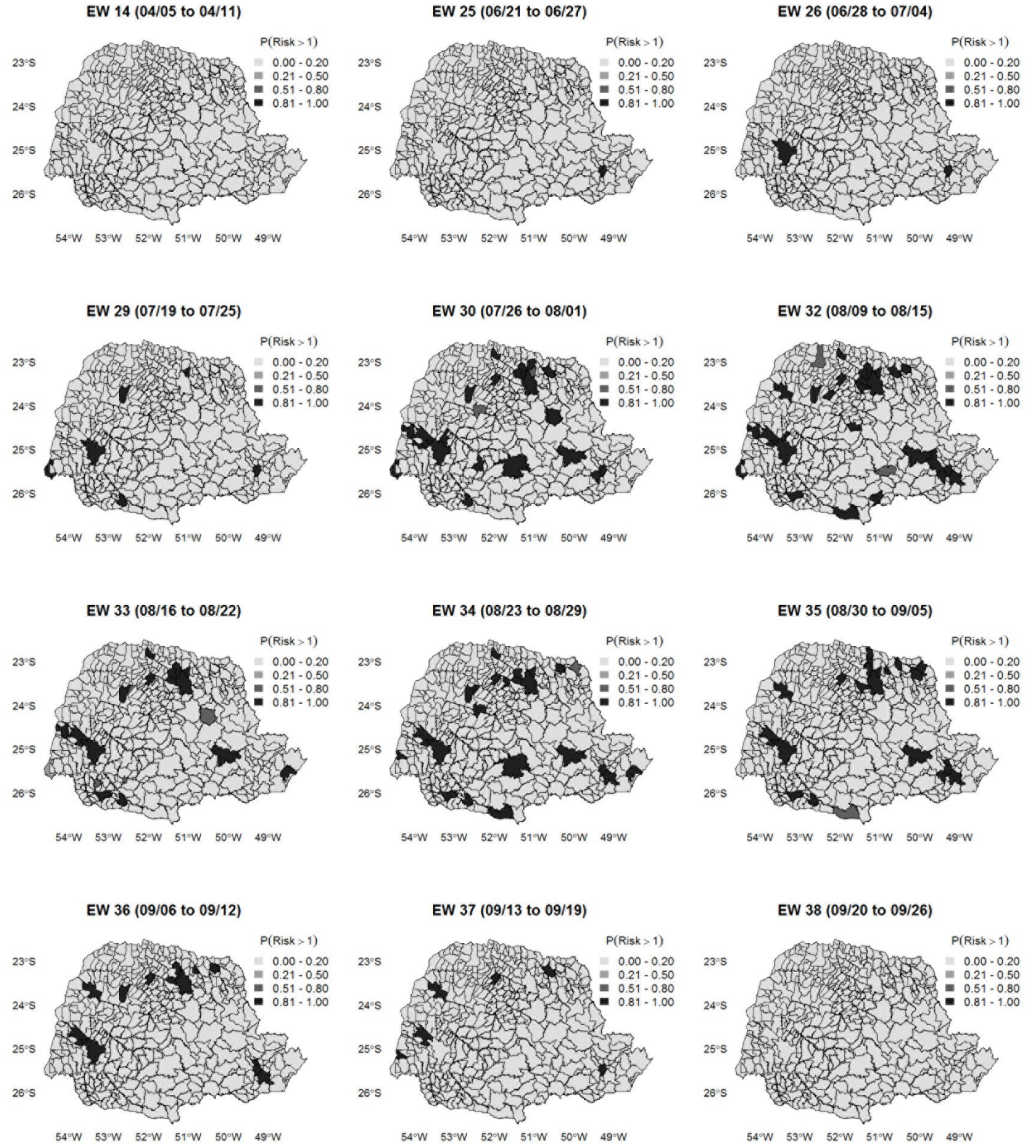
Influenza A (H1N1) risk probability map. Map of the posterior probabilities of influenza A (H1N1) risk exceeds 1 during selected epidemiological weeks in the state of Paraná.

Fig 7 shows the excess of influenza risk, that is, in the probability *P*(*Risk* > 1). In EW 14 (04/05 to 04/11), which marked the beginning of the study period, the probability of excess risk was considered low for all municipalities of Paraná. However, in EW 25 (06/21 to 06/27), a period of high risk of transmission, there was an increased risk of influenza in the state capital, Curitiba. From EW 29 (07/19 to 07/25), the week following the official announcement of sustained transmission of the disease, there were significant increases in the risk of influenza A for two of the most populous municipalities in the western region of Paraná, two municipalities in the southwest and northwest regions, and another in the north-central region, in addition to Curitiba, which maintained a high risk. Since then, other areas of dissemination appeared, increasing the risk of spread of the disease, especially to neighboring municipalities. From EW 32, there was a slow and progressive reduction of the risk of influenza A (H1N1). At EW 38, the risk of disease transmission was considered low for all municipalities.

**Fig 8:**
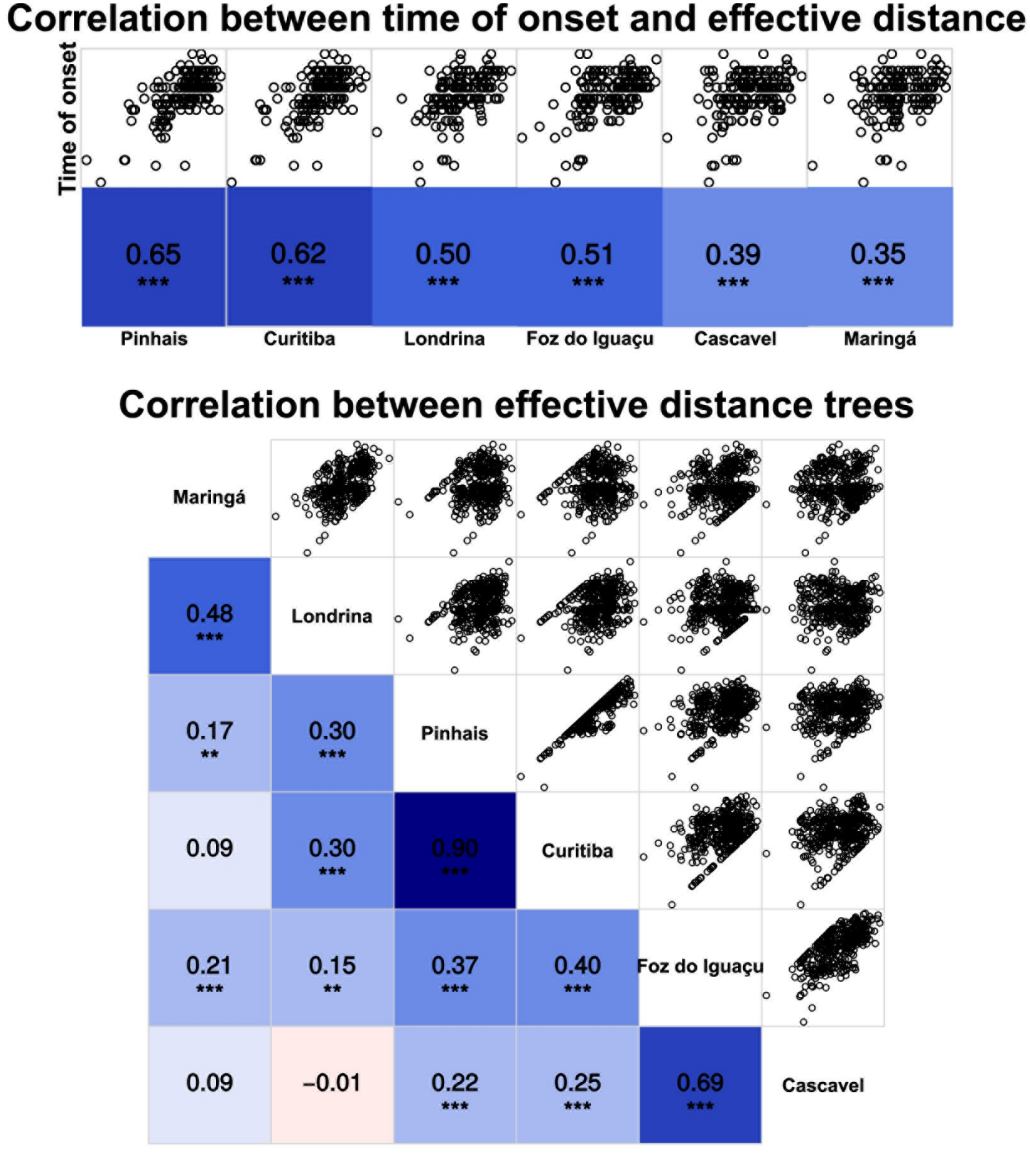
Correlation chart. Correlation between the arrival times of the epidemic and the effective distance for candidate municipalities for the starting point of influenza A (H1N1) in Paraná in 2009.

Fig 8 illustrates the analysis of candidate municipalities as the starting points of the spatial diffusion process of influenza A (H1N1) in Paraná in 2009. The values in the center of the blue squares in the first column of the matrix represent the Spearman correlation coefficients between the time of arrival of the epidemic and the effective distance. The upper triangular part of the matrix shows the scatter plots which produced the correlation coefficients for each of the candidate municipalities. As expected, regardless of origin the greater the effective distance of the candidate municipality, the longer the arrival time of the epidemic. It is also easily observable that the municipalities of Pinhais and Curitiba presented the highest correlations and, therefore, are strong candidates for the origin of the process of diffusion of influenza. It is noteworthy that the connectivity matrices and, consequently, the effective distances for both municipalities, are quite similar (correlation equal to 0.94). This is due to the fact that Pinhais has a very strong connection to Curitiba, the State Capital. The number of individuals frequently traveling to Curitiba is 20 times larger than to the second main destination, and concentrates almost 84% of the flow from Pinhais. Therefore, the paths from Pinhais to other municipalities are mostly governed by those from the Capital.

## Discussion

This study aimed to characterize the spatiotemporal distribution of the influenza A pandemic in the Brazilian state of Paraná from the introduction of the H1N1 virus until the end of the epidemic period in 2009, aiming to understand the process of diffusion in the state and to identify risk factors for occurrence and intensity. The results indicated that interventions with a focus on municipalities with greater flow and density of people, especially those with higher HDI and the presence of municipal air and road transport, could play an important role in mitigating the impact of future influenza pandemics on public health.

One limitation of conventional regression models for counting data is that both zeros and non-zero (positive) observations are assumed to come from the same data generation process. The application of the two-stage model for infectious disease surveillance data is flexible in this regard, allowing the identification of different risk factors for the occurrence and intensity of disease due to the presence of excess zeros in the data set. The proposed methodology for estimation allowed for rapid and efficient implementation of the spatiotemporal model, which showed different patterns throughout the epidemiological weeks for the occurrence and intensity of influenza transmission.

Pendular migration data have been increasingly used to describe population mobility in epidemic models [21–28]. The present study showed that the pendular migration is significantly associated with the chance of occurrence of influenza, such that municipalities with greater flow of people have a greater chance of occurrence of the disease. Charaudeau et al. [29], using data from epidemics of influenza-like illness in France, also showed that pendular migration is highly correlated with the spread of disease.

Paraná is a border state and thus an important gateway into the country. It is known that the first cases of influenza A (H1N1) in Brazil were imported from countries that already had sustained transmission of the disease [4]. The pandemic in 2009 clearly showed the importance of air transport as a means of spreading influenza virus [30–33]. In our study, the existence of air transport contributed significantly to the process of dissemination of the disease (in terms of the chance of occurrence). According to Askling and Rombo [7], influenza has always been related to travel patterns and, currently, disease epidemiologists commonly include air travel as an essential part of the dissemination process.

With the rapid growth of public transport infrastructure and increased socioeconomic activities, travel patterns have become important issues in the prevention of airborne infectious diseases such as influenza, especially during the introduction period [34]. Obviously, the presence of airports and high densities of transport routes coincide with more developed areas, that is, those with greater population density and access to health [34].

In a socioeconomic context, HDI data allows an ordering among municipalities and provides a means of determining the level of development of each region. In this study, the HDI was significant indicator of a greater chance of occurrence and intensity of influenza A (H1N1) transmission in more populous municipalities, that is, those with higher HDIs. Xiao et al. [35] analyzed the spatiotemporal spread of influenza A (H1N1) in Changsha, China in 2009 and the factors that influenced the diffusion process. They showed that the regions with the highest incidence rates were concentrated in more economically developed areas, such as cities and economic districts.

Population density in Paraná is very heterogeneous, with the lowest density in the Alto Paraiso municipality, with 3.31 inhabitants per km^2^, while Curitiba, the capital and most populous municipality, has an average of 4,024.84 inhabitants/km^2^. There was a significant increase in the risk of influenza A (H1N1) in more densely populated municipalities. Lopez et al. [36] described the initial outbreak of the new virus in Mexico City and showed that the largest numbers of confirmed cases of the disease were observed in more populous districts. They concluded that high population density in the district of Iztapalapa contributed to the spread of the epidemic. A study by Fang et al. [34] also indicated that population density contributed to the spread of the influenza A (H1N1) epidemic in China. Another study carried out in China found that the 2009 influenza A pandemic affected heavily populated cities more than others [37].

Since transmission of influenza occurs through contact, secretions, and inhalation of aerosol particles, contamination may also occur within public transport facilities and passenger embarking stations [38, 39]. In this study, the existence of municipal road transport was positively and significantly associated with the risk of influenza A (H1N1). According to Freedman and Leder [40], transport networks by virtue of direct contact with large numbers of individuals allow greater spread of pathogens and make populations exposed to public transport systems most susceptible to infection. Maliszewski e Wei [41] also found that public transport use rates were significantly and positively associated with hospitalization rates related to influenza A (H1N1) 2009 in California in the United States.

In addition to sociodemographic factors that may favor the establishment and maintenance of the virus in individuals, it is also known that influenza virus is better adapted to replicate at temperatures lower than the average for most common infectious agents [42]. Temperature may also directly or indirectly influence human behavior with regard to contact rate and aggregation patterns in ways that increase the risk of disease transmission [43]. Our results showed that maximum temperature in the week prior to case reports significantly affected the risk of influenza for several weeks. We found evidence early on that an increase in maximum temperature favored increased risk of influenza, possibly reflecting patterns of human aggregation. This counterintuitive result may be partially explained by the fact that with lower temperatures, people leave the home less, which may reduce the intensity of transmission. In Brisbane, Australia, Hu et al. [44] observed significant increases in the incidence of influenza A (H1N1) associated with decreases in maximum temperature with a one-week lag. Lopez et al. [45] also found a negative correlation between temperature and the prevalence of influenza A (H1N1) in a semi-arid region in India. In this study, a significant increase in the risk of influenza transmission was associated with a drop in temperature from weeks 21 to 28. From an ecological point of view, few authors have verified the impact of climatic factors on influenza in tropical regions, where temperatures and relative humidity are generally higher [46–48]. In addition, to our knowledge there are no studies that consider that the effect of temperature on the risk of influenza can vary over time, as proposed in this work.

Identifying the starting point of influenza epidemics is fundamental for the development of timely intervention strategies and to predict the spread to other municipalities. The municipalities of Pinhais and Curitiba proved to be strong candidates for the origin of spatial diffusion of influenza. According to the Gazeta do Povo newspaper on July 18, 2009 (page 4), “a 24-year-old man may have been the first victim of influenza A (H1N1), known as swine flu, in the Curitiba region”. The Hospital and Maternity Ward of Pinhais was quarantined in July 2009 for disinfection, and four employees who lived with infected patients were given temporary leave as a means of prevention [6]. From a methodological point of view, this result showed how complex spatiotemporal patterns of propagation can become surprisingly simple, if measures of conventional geographical distances are replaced by the probabilistic concept of effective distance.

An understanding of the factors influencing disease occurrence will strengthen surveillance actions in the contention phase in order to reduce the probability of transmission to other municipalities. The results showed distinct dissemination foci in several regions of Paraná, indicating that introduction of the virus was likely mediated by air travel, and that disease propagation was promoted in areas with greater flow of people and socioeconomic activity, allowing diffusion and persistence throughout the state.

The present study does have a few limitations. First, a common limitation in ecological studies is the inability to control for effects of confounding factors at the individual level. A second limitation is probable sources of information bias, including under-reporting in asymptomatic persons, infected persons that do not seek medical help, or as a result of changes in the reporting system that occurred after July 1, at which point diagnostic efforts were focused on cases of severe acute respiratory syndrome, risk groups, and outbreaks. Collected during an emergency scenario and restricted to clinical cases, the data do not provide a full picture of the influenza transmission landscape. It is possible that detection differed between municipalities. On the positive side, previous analysis showed that Paraná had the most sensitive surveillance system among the Brazilian states, with significantly higher reporting rate of mild cases [5]. Within the state the three metropolitan regions, Curitiba, Foz do Iguaçu and Londrina-Maringá, presented similar mild-to-severe ratios, suggesting a certain level of homogeneity in the notification pattern. Finally, due to lack of available information the temporal data associated with some explanatory variables differs from that of the dependent variable. For example, the reported cases of influenza refer to 2009, while the covariates poverty rate, municipal HDI, and migration data were derived from the 2010 Census. It is, however, reasonable to assume that sociodemographic information did not undergo significant changes over the course of a single year.

Despite these limitations, this study provided a comprehensive ecological view of the process of spatiotemporal diffusion of pandemic influenza A (H1N1) in Paraná in 2009. These results provide important information on the origin and spread of influenza in the state of Paraná, and may help to identify priority areas for surveillance and the establishment of strategic measures for disease prevention and control. The application of the proposed model also allows identification of epidemiological weeks with an excessive chance of influenza occurrence, and can be used as reference criteria for defining immunization campaign schedules.

## Supporting information

S1 Table Dataset used for analysis. Epidemiological, Sociodemographic and Climate Data.
(CSV)

S1 Fig Goodness of fit of the ZAP model. Posterior median (solid line), 95% credible intervals (dashed line) of the fitted values and observed values (filled circle) throughout the epidemiological weeks for 4 municipalities.
(TIFF)

S2 Fig Goodness of fit of the Binomial model. Predicted probabilities (solid line) of influenza occurrence and observed values (filled circle) throughout the epidemiological weeks for 4 municipalities.
(TIFF)
