# Supplementary figures and images for: Spatiotemporal diffusion of influenza A (H1N1): Starting point and risk factors
Source: PLoS One. 2018 Sep 4;13(9):e0202832. doi: 10.1371/journal.pone.0202832 (PMC6122785; doi:10.1371/journal.pone.0202832)

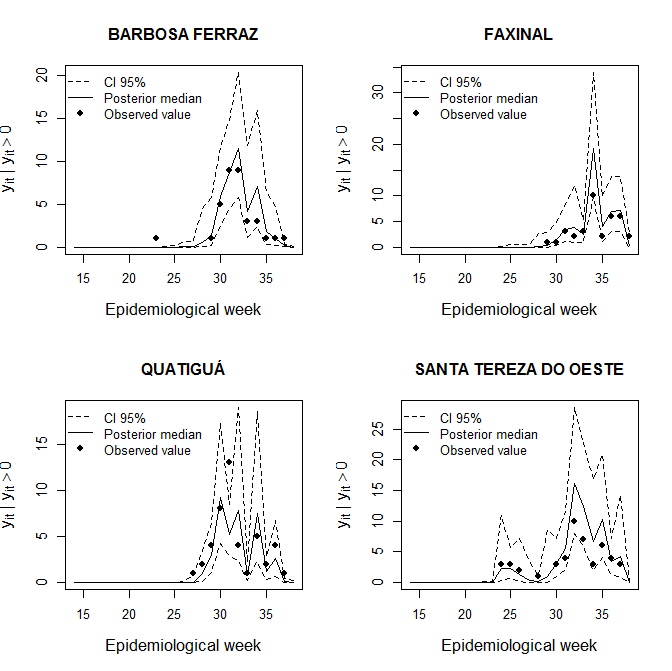

Supplement: S1 Fig — Posterior median (solid line), 95% credible intervals (dashed line) of the fitted values and observed values (filled circle) throughout the epidemiological weeks for 4 municipalities. (TIFF) [file pone.0202832.s002.tiff]

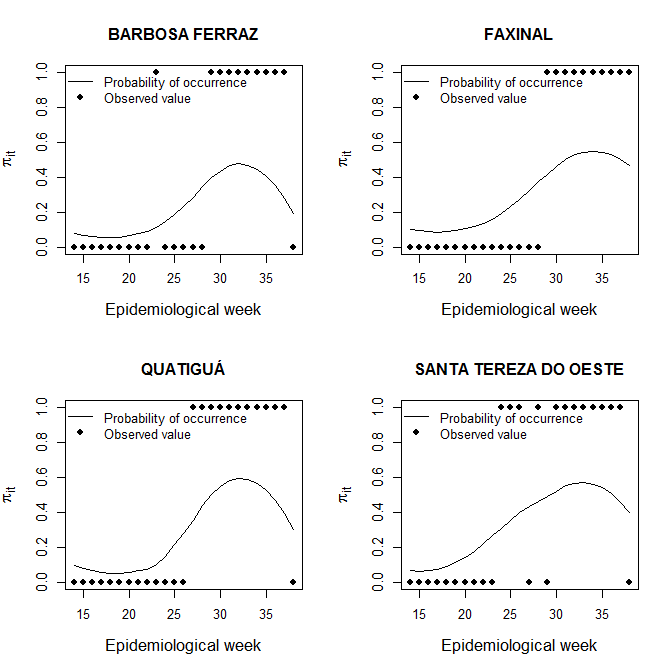

Supplement: S2 Fig — Predicted probabilities (solid line) of influenza occurrence and observed values (filled circle) throughout the epidemiological weeks for 4 municipalities. (TIFF) [file pone.0202832.s003.tiff]
